# Large-scale, thick, self-assembled, nacre-mimetic brick-walls as fire barrier coatings on textiles

**DOI:** 10.1038/srep39910

**Published:** 2017-01-05

**Authors:** Paramita Das, Helga Thomas, Martin Moeller, Andreas Walther

**Affiliations:** 1 DWI – Leibniz-Institute for Interactive Materials, RWTH Aachen University, Forckenbeckstr. 50, 52056 Aachen, Germany

## Abstract

Highly loaded polymer/clay nanocomposites with layered structures are emerging as robust fire retardant surface coatings. However, time-intensive sequential deposition processes, e.g. layer-by-layer strategies, hinders obtaining large coating thicknesses and complicates an implementation into existing technologies. Here, we demonstrate a single-step, water-borne approach to prepare thick, self-assembling, hybrid fire barrier coatings of sodium carboxymethyl cellulose (CMC)/montmorillonite (MTM) with well-defined, bioinspired brick-wall nanostructure, and showcase their application on textile. The coating thickness on the textile is tailored using different concentrations of CMC/MTM (1–5 wt%) in the coating bath. While lower concentrations impart conformal coatings of fibers, thicker continuous coatings are obtained on the textile surface from highest concentration. Comprehensive fire barrier and fire retardancy tests elucidate the increasing fire barrier and retardancy properties with increasing coating thickness. The materials are free of halogen and heavy metal atoms, and are sourced from sustainable and partly even renewable building blocks. We further introduce an amphiphobic surface modification on the coating to impart oil and water repellency, as well as self-cleaning features. Hence, our study presents a generic, environmentally friendly, scalable, and one-pot coating approach that can be introduced into existing technologies to prepare bioinspired, thick, fire barrier nanocomposite coatings on diverse surfaces.

Fire remains one of the largest reasons for non-natural fatalities and causes dramatic loss of property and gross national product in the range of billion euros every year. Cotton, a natural textile fiber, is extensively used in everyday products, such as clothing, furniture, and industrial products. Yet, cotton exhibits a low limiting oxygen index, ease of ignition, and high flammability^1^. Many attempts have been made to make fire-retardant cotton fabric^2^,^3^. Most effective present-day fire-retardant materials use heavy metals^4^,^5^,^6^,^7^ or bromine^8^,^9^-containing species to efficiently arrest fires. In the light of environmental and health concerns, and upcoming legislation restrictions regarding brominated fire retardants^9^,^10^,^11^, it is thus very important to develop new procedures for intrinsically fire retardant bulk materials, as well as, for fire-retardant and fire-blocking coatings, at best using environmentally benign materials and processes^12^,^13^. The combination of tailored polymers with inorganic nanoparticles, e.g. nanoclays^14^,^15^,^16^,^17^,^18^,^19^,^20^,^21^,^22^,^23^ or carbon nanotubes^24^,^25^,^26^,^27^, glass fabric^28^, polyhedral silsesquioxanes (POSS)^29^,^30^, layered double hydroxides^31^,^32^,^33^,^34^, providing oxygen barrier properties and mechanical strength, preventing dripping of hot, molten plastics, and enabling an intumescent, self-expanding foam-like heat barrier behavior is a most promising approach for fire-retardant coating materials^35^. Aiming at a quantitative understanding in such materials requires approaches towards structurally well-defined surface coatings, in which benefits in the fire behavior can explicitly be linked to defined composition and architecture.

Interesting progress towards nanostructured, fire-resistant coatings has been reported for ultrathin coatings based on step-wise, layer-by-layer (LbL) deposition of different polymers and nanoclay^22^,^36^,^37^,^38^,^39^,^40^,^41^,^42^, in which substrates are alternatingly dipped into polymer and nanoclay to build up multilayers of organic and inorganic phases. Even though attractive fire barrier properties could be shown, the process requires several deposition steps needed to reach the desired properties (e.g., 20–40 deposition steps)^43^,^44^,^45^,^46^,^47^,^48^. Recently, a high efficiency LbL coating based on starch showed self-extinguishing behavior of cotton only after 4–8 deposition steps^48^. However, it is yet difficult to reach thicknesses beyond tenth or hundreds of nanometer via LbL, and the technique remains sequential, and laborious. Hence, there is still a tremendous need to develop simple, fast and scalable procedures to prepare thick coatings with tailored nanostructure based on readily available constituents and presenting excellent fire barrier properties.

Here, we show a one-pot, single-step self-assembly approach to prepare thick, bioinspired, layered, hybrid brick-wall coatings with well-defined nanostructure on cotton textiles, as formed through self-assembly of tailored mixtures of sodium carboxymethyl cellulose (CMC) and montmorillonite (MTM) nanoclay at high fractions of the inorganic component. The coating thickness on the textile can be tailored using different CMC/MTM concentrations in the coating bath. We study the fire barrier and fire retardancy properties as a function of coating thickness by themogravimetric analysis (TGA), fire break-through test, vertical flame test (VFT), thermal imaging using forward looking infrared (FLIR) camera, and cone calorimetry. In addition, we modify the surface of the fire-retardant coating to impart amphiphobic and self-cleaning features. Hence, this study demonstrates a scalable process, that can be introduced into existing coating technologies to provide bioinspired, multifunctional, fire barrier nanocomposite coatings on textile, using benign water-based room temperature processing.

## Results

### Self-assembled fire-barrier coatings on textile and structural characterization

**Figure 1:**
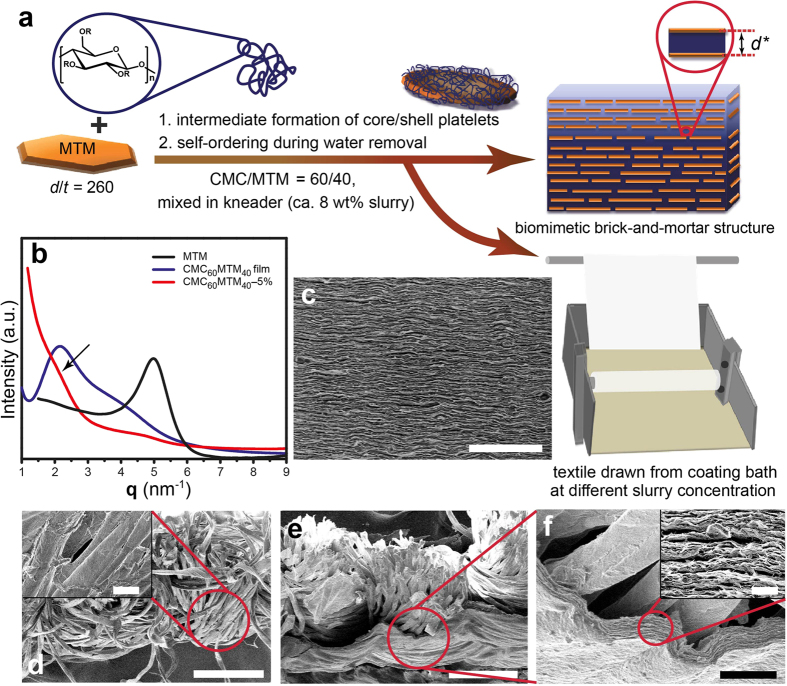
Preparation of bioinspired nanocomposite coatings and structural characterization. (**a**) Schematic of the self-assembled nanocomposite films based on CMC polymer, (R = H or CH_2_-COONa) and montmorillonite (MTM) natural nanoclay via concentration-induced self-assembly of polymer-coated nanoclay platelets with intrinsic hard/soft architecture, and coating of textiles from different concentrations of CMC/MTM = 60/40 w/w dispersions at 10 mm/min. (**b**) XRD of the pure MTM film, CMC_60_MTM_40_ film, and CMC_60_MTM_40_-5% coated fabric. The arrow shows the appearance of a diffraction hump for the coated textile. (**c**–**f**) SEM images of the cross-sections of (**c**) CMC_60_MTM_40_ film (scale bar 5 μm), (**d**) CMC_60_MTM_40_-1% (scale bar 200 μm, inset: scale bar 10 μm), (**e**) CMC_60_MTM_40_-5% (scale bar 200 μm), and (**f**) high magnification image of the CMC_60_MTM_40_-5% (scale bar 20 μm). CMC_60_MTM_40_-1% exhibits only a conformal coating of the fiber (**d**, inset, and Supplementary Fig. S1), and CMC_60_MTM_40_-5% shows the ordered layered structure of the nacre-mimetic coating (**f**, inset: scale bar 2 μm).

The general strategy for the preparation of self-assembled, layered, bioinspired nanocomposite coatings on cotton textiles is shown in Fig. 1a. We start with the preparation of CMC/MTM dispersions by kneading a paste at CMC/MTM = 60/40 (w/w) at ca. 8 wt% solid content in water. This allows the formation of homogeneous, concentrated slurries of both components due to well-balanced polymer/nanoclay interactions – even at high solid content. Well-defined core/shell nanoplatelets are formed during this process by adsorption of CMC onto MTM nanoplatelets via hydrogen bonding and the hydrophobic effect^49^. During water evaporation, these slurries self-assemble into well-defined, hybrid, nanostructured brick-walls, with well-ordered alternating hard and soft layers as pre-encoded by the core/shell structure of the polymer-coated nanoclay^49^,^50^,^51^. For applying the coating, the cotton textiles are passed through a bath containing different concentrations of the CMC/MTM (60/40 w/w) hydrocolloid dispersion (1, 1.75, 2.5, 5 wt%, after dilution from the initial paste) at 10 mm/min, and subsequent drying. We denote the specimens as CMC_60_MTM_40_-x%, where the subscripts denote the weight fractions of both components and ‘x%’ denotes the concentration of the coating dispersion in wt%. Reasonably, we can expect that the coating thickness (weight/area) depends on the concentration of the coating dispersions and that a morphological transition may occur starting from coating of individual fibers at low concentration/viscosity to the presence of a fully coated film at high concentration/viscosity. For comparison, we also prepare a nacre-mimetic nanocomposite film by solution casting of a CMC/MTM dispersion in a petri dish. This allows characterizing the nanocomposite structure in a straightforward manner and serves as a comparison to structures created on textiles. The structural characterizations of the nanocomposite film, as well as, CMC_60_MTM_40_-1%, and CMC_60_MTM_40_-5% coated fabrics are displayed in Fig. 1b–f. X-ray diffraction (XRD) of the nanocomposite film shows a primary diffraction peak (q*) in the range of 1.7–2.3 nm^−1^, corresponding to gallery spacing in the range of 3.7–2.7 nm. Although the diffractogram of the coated fabric does not present very sharp peaks due to the undulated surface of the textile, it still clearly shows a hump (arrow in Fig. 1b) in the same range, thus confirming the successful formation of nacre-mimetic structures on the fabric. Weak and broad humps in both diffractograms appear around 3.5–5.3 nm^−1^, which is the 2q* peak corresponding to the higher-order peaks characteristic of a lamellar morphology. The dominant peak of pure MTM is strongly diminished, indicating the achievement of high levels of delamination in the kneading process.

Further investigation of the morphology by SEM confirms the desired well-aligned layered structure in the films and coatings. Notably, the high magnification SEM image of CMC_60_MTM_40_-5% (Fig. 1f, inset) clearly shows that in addition to coating of individual fibers, a continuous film with 8.5 ± 3 μm thickness remains on the surface of the whole fabric when coating using high concentrations (Fig. 1e).

Such a film is absent for lower slurry concentrations in the coating bath (e.g. 1 wt%), where only a conformal coating of the fibers exists (Fig. 1d, inset and Supplementary Fig. S1). Formation of such a film is related to the high viscosity of the slurry and to the coating procedure. It is an important difference to polymer/nanoclay multilayers formed by LbL, which can only form conformal coatings on fibers^38^,^52^,^53^.

**Figure 2:**
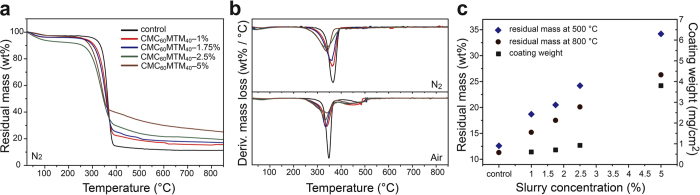
Thermal and thermo-oxidative stability of CMC_60_MTM_40_-coated fabrics and the control. (**a**) TGA plot of the control and CMC_60_MTM_40_-1%, 1.75%, 2.5%, and 5% coated fabrics in N_2_. (**b**) DTGA plots (top) in N_2_ and (bottom) in air. (**c**) The average coating weight added on the textile from different slurry concentrations (calculated gravimetrically), and the residual mass calculated from TGA at 500 and 800 °C in N_2_ atmosphere for all samples.

The thermal and thermo-oxidative stability of the coated fabrics and control are examined by thermogravimetric analysis (TGA) in both N_2_ and air atmosphere from 25 to 850 °C at 10 °C/min as shown in Fig. 2.

The coated fabrics exhibit similar decomposition curves like the control, but they undergo a slightly earlier onset of degradation (Fig. 2a,b). Differential thermogravimetric analysis (DTGA) plots in Fig. 2b show that pure cotton (control) begins to degrade around 280 and 275 °C in N_2_ and air, respectively. The initial minute weight loss at ca. 110 °C is due to the removal of moisture. The major weight loss occurs between 280 to 400 °C in both atmospheres resulting from the decomposition and dehydration of the cellulose leading to thermally stable residues. The second weight loss between 400 to 530 °C in air corresponds to the further oxidation of the char to produce CO and CO_2_^54^. Despite having an earlier onset, CMC_60_MTM_40_-coated fabrics undergo slower degradation with increasing the slurry concentration as seen from the smaller and wider peak in the DTGA plots when going from CMC_60_MTM_40_-1% to CMC_60_MTM_40_-5% (Fig. 2b)^55^. This slower rate of thermal degradation arises from the highly ordered, layered arrangement of the large MTM nanoplatelets (aspect ratio = 260^56^) inside the CMC/MTM coating. On one hand, this layered nanocomposite structure provides thermal insulation and reduces the heat transfer into the cotton fabric, and on the other hand, it lowers oxygen diffusion due to the tortuous pathways (oxygen barrier property = 0.022 cm^3^ · mm·m^−2^ · day^−1^·atm^−1^ at 50%RH and 0.115 cm^3^ · mm·m^−2^·day^−1^·atm^−1^ at 80%RH)^54^,^57^. The residual mass of the coated fabrics at 500 °C (after the first step of degradation), and at 800 °C (almost at the final stages of degradation) in N_2_ increases with increasing slurry concentrations (Fig. 2c). This indicates a higher inorganic content originating from thicker coatings. The average weight per area added to the fabric ranges between 0.6 and 3.8 mg/cm^2^ with increasing the concentration from 1 to 5 wt% in the coating bath. The values reflect the tendency found in SEM, where thinner conformal coatings on individual fiber are observed at lower slurry concentrations, while a continuous thicker film is formed in addition to coating of individual fibers at higher concentrations (Fig. 1d–f). Moreover, such mechanically robust CMC/MTM coating on cotton also provides better mechanical integrity to coated fabrics as compared to the uncoated ones^49^,^50^. We observed a stiffening of the coated cotton fabrics with increasing the coating concentration from 1 to 5 wt% due to higher inorganic content and coating thickness.

### Fire-barrier and fire-retardancy test

**Figure 3:**
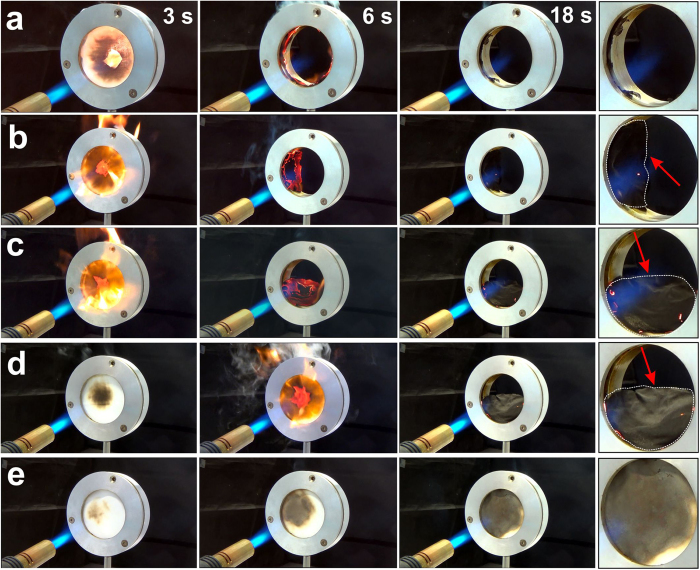
Fire break-through test. Photographs of (**a**) the uncoated control, (**b**) CMC_60_MTM_40_-1%, (**c**) CMC_60_MTM_40_-1.75%, (**d**) CMC_60_MTM_40_-2.5% and (**e**) CMC_60_MTM_40_-5% at times indicated during exposure to a high temperature torch flame (ca. 1750 °C) at nearly 90° angle and 130 mm away from the sample. Right column: Shrinkage of the coated fabrics during charring. Sample edges are marked in dotted white line.

Next, we study the ability of the textiles to withstand direct high intensity flames from a short distance (130 mm) using a fire break-through test. All samples were fixed in a circular holder with ca. 7 cm diameter and then exposed to a high temperature torch at nearly 90° angle. Figure 3 displays time-lapse photographs taken during the test. The control catches fire within 3 s, and is consumed completely after 18 s without leaving any residue in the holder. In contrast, all coated fabrics char into a solid barrier material resisting long term exposure to direct flames. Most coated fabrics undergo shrinkage during charring in the direct flame, and get loose from the sample holder (see inset of Fig. 3b–d). We believe that contraction and distortion should be less relevant for larger specimens.

An increase of the coating thickness limits this behaviour, and CMC_60_MTM_40_-5% chars into a shape-persistent, solid barrier, which completely restricts the fire from breaking through (Fig. 3e). Figure 3 further shows that the time to catch fire gradually increases with increasing slurry concentration, and the best fire barrier is observed for CMC_60_MTM_40_-5%, which chars but does not support any flame even after direct exposure for 60 s.

**Figure 4:**
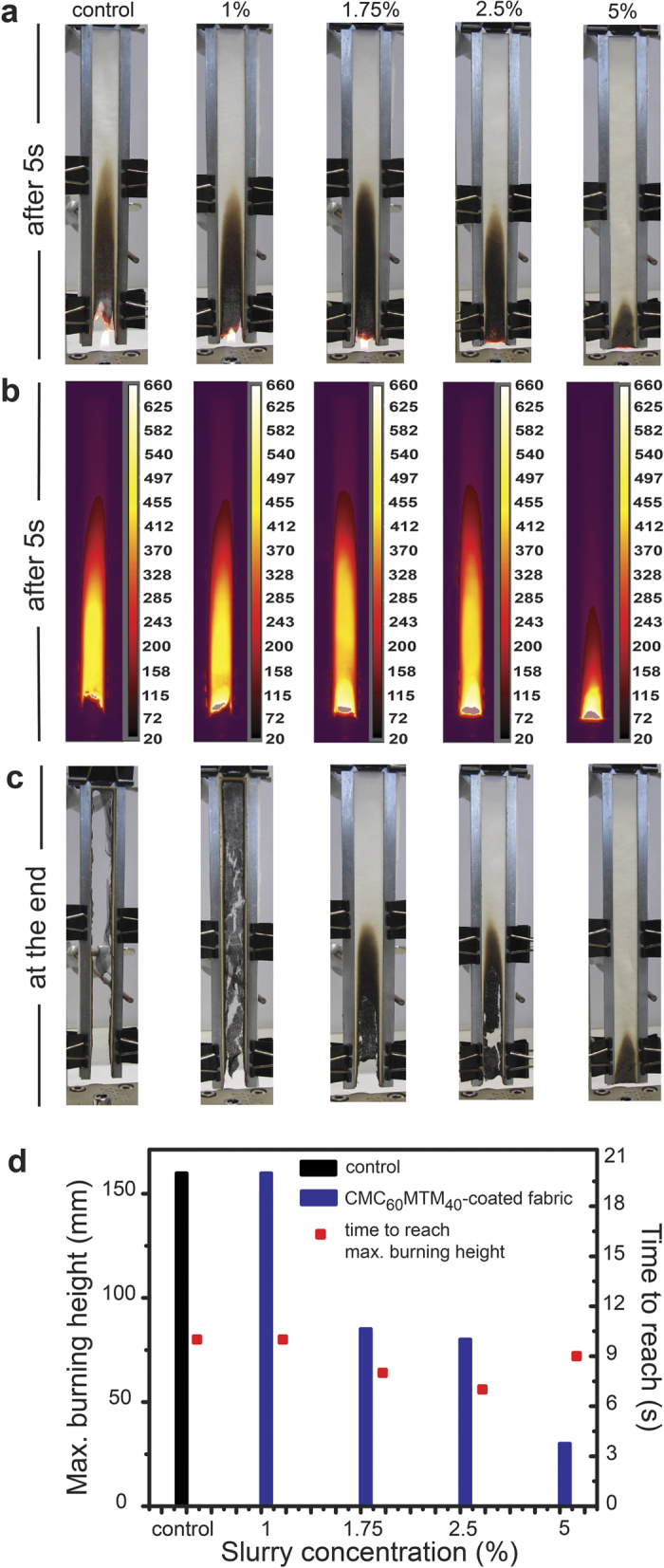
Bench-scale vertical flame test (VFT) analysis. (**a**) Photographs from VFT of CMC_60_MTM_40_-coated fabrics and the control after 5 s of ignition. (**b**) Corresponding FLIR images after 5 s of ignition with a color scale from 20 to 660 °C. (**c**) Photographs of VFTs at the end of burning. (**d**) Maximum burning height and the minimum time needed to reach that height for CMC_60_MTM_40_-coated fabrics and the control.

To study flame retardancy and self-extinguishing behavior, we performed bench-scale vertical flame test (VFT) on specimens with dimensions of 160 mm × 12.5 mm (L × W) in analogy to EN ISO 11925-2. The flame was kept at the bottom of the sample and ignited for 5 s. A forward looking infrared camera (FLIR) allows monitoring the spatiotemporal temperature profiles and standard video monitors the general behavior. Figure 4 shows the results of bench-scale vertical flame test. The optical photographs and FLIR images after 5 s of ignition, and optical photographs at the end of burning are shown in Fig. 4a–c. The photographs after 5 s of ignition show that the flame is much brighter and stronger in case of the control, but becomes less vigorous for fabrics coated in higher slurry concentrations and thus having higher thicknesses. Figure 4d further depicts that the maximum burning height of the samples reached by either flame propagation or smoldering decreases with increasing coating thickness.

**Figure 5:**
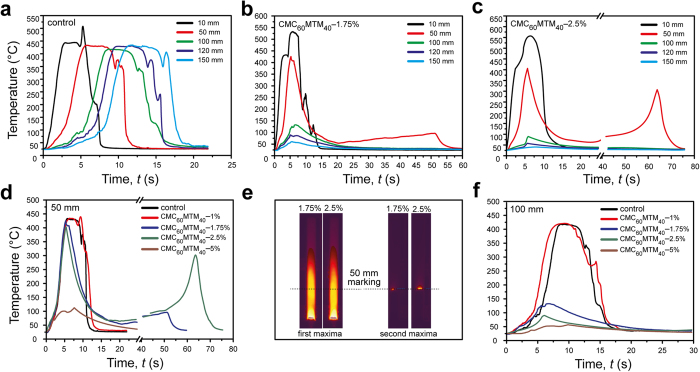
Temperature vs. time plots of the CMC_60_MTM_40_-coated fabrics and the control at different sample height during bench-scale VFT. (**a**–**c**) Temperature profiles of the control, CMC_60_MTM_40_-1.75%, and CMC_60_MTM_40_-2.5% at 10, 50, 100, 120 and 150 mm sample height. (**d**,**f**) Comparative study of the temperature profiles of all CMC_60_MTM_40_-coated fabrics at (**d**) 50 and (**f**) 100 mm. (**e**) FLIR images of CMC_60_MTM_40_-1.75% and CMC_60_MTM_40_-2.5% corresponding to the first temperature peak at ca. 5 s (hot gas) and second temperature peak at 50 s and 65 s, respectively, (glowing frontier), (temperature scale bar same as in Fig. 4b).

The control burns immediately and is consumed completely within 19 s after ignition (Fig. 5a). CMC_60_MTM_40_-1% still allows flame propagation and complete burning, yet it shows a significant amount of coherent char with some slits at the end. The behavior of CMC_60_MTM_40_-1.75% and CMC_60_MTM_40_-2.5% is different. Those are not consumed completely, but burn up to half of the sample height. The flame reaches a maximum height of ca. 85 and 80 mm in 8 and 7 s after ignition for CMC_60_MTM_40_-1.75% and CMC_60_MTM_40_-2.5%, respectively, leaving behind a broken solid char due to the presence of higher coating thickness. Although they self-extinguish right after removal of the direct flame, a glowing frontier persists, which propagates very slowly within the burning height (ca. 50 mm, Fig. 5e) and vanishes completely at 50 and 90 s after ignition for CMC_60_MTM_40_-1.75%, and CMC_60_MTM_40_-2.5%, respectively, (Fig. 5d). CMC_60_MTM_40_-5% performs extremely well in terms of fire retardancy. It self-extinguishes once the direct flame is removed, and smolders up to a maximum height of 30 mm in 9 s after ignition. It does not support either flame or glow propagation, and forms a continuous and intact solid charred residue due to the highest coating thickness. Dripping is not observed in any sample.

The spatiotemporal FLIR imaging allows deeper insights into the thermal behavior and flame propagation. While stills from the FLIR videos after 5 s of ignition are shown in Fig. 4b, the full range temperature vs. time profiles of the samples are shown in Fig. 5. First, we discuss the temperature profiles at different sample heights (from 10 to 150 mm) of the control and the CMC_60_MTM_40_-coated fabrics from two intermediate slurry concentrations, serving as the most instructive examples (CMC_60_MTM_40_-1.75% and CMC_60_MTM_40_-2.5%; Fig. 5a–c). Afterwards, we focus on the comparison of the temperature profiles at two intermediate sample heights (50 and 100 mm) for all samples (Fig. 5d–f).

The temperature profiles of the control in Fig. 5a reveal a consistent shift of the traces with increasing sample height. They exhibit temperatures around 400–500 °C during passage of the flame front. The behavior is different for CMC_60_MTM_40_-1.75% and CMC_60_MTM_40_-2.5% samples. Here, the flame stops before reaching 100 mm (max. flame height ca. 85 mm), and therefore, the temperature profiles at 100, 120 and 150 mm are essentially flat against time, except for the initial 5–15 s, where some hot gases from the flame and the sample pass the markings. At 10 mm, both samples show the initial burning process initiated by the exposure to the direct flame. At 50 mm, interestingly, two maxima can be identified (Fig. 5b–d). The first maximum corresponds to the initial flame exposure and burning of the sample, and originates from the ascent of hot gases. While, the second maximum at comparatively lower temperature arises from a delayed glow frontier, which smolders slowly across the sample until the glow vanishes. The glow frontier stops just below the 50 mm marking (lower temperature increase) in CMC_60_MTM_40_-1.75%, while it stops just on or above the marking in CMC_60_MTM_40_-2.5%, causing higher temperature rise in the second maxima as compared to CMC_60_MTM_40_-1.75%. The photographs in Fig. 5e serve to illustrate the two maxima at different times. At 100 mm the two maxima are absent as the glowing frontiers vanish before reaching this height (Fig. 5f). The comparison of all coated samples also shows that all coated fabrics show a significant drop in temperature at a sample height 100 mm or higher, except for CMC_60_MTM_40_-1%, which does not differ much from the control. Similar to the control, CMC_60_MTM_40_-1% is consumed completely (yet with higher char), due to the comparably thin coating.

**Figure 6:**
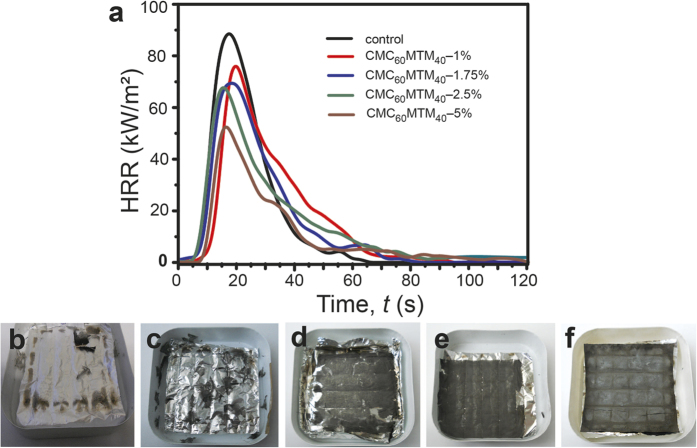
Cone calorimetry measurements of the CMC_60_MTM_40_-coated fabrics and the control. (**a**) Heat release rate of the CMC_60_MTM_40_-coated fabrics for all slurry concentrations and the control. (**b**–**f**) Photos of the residues of the coated fabric and the control after burning in cone calorimetry, (**b**) control, and (**c**–**f**) CMC_60_MTM_40_-1%, 1.75%, 2.5% and 5%, respectively.

To better predict the combustion behavior of the materials in real fire situation, we further estimate the heat release rate (HRR) and flammability of the coated fabrics and the control using cone calorimetry under a constant heat flux of 50 kW/m^2^ (Table 1 and Fig. 6a–f)^58^. Although the time to ignition (TTI) remains similar for all CMC_60_MTM_40_-coated fabrics and the control, increasing the coating thickness for the coated fabrics results in continuous reduction in peak heat release rate (pkHRR), total heat release (THR), and total smoke release (TSR). Furthermore, the heat profiles are more extended in the time axis for increasing coating thickness. This demonstrates that all CMC_60_MTM_40_-coated fabrics exhibit significantly higher fire-retardancy as compared to the control^55^. The CMC_60_MTM_40_-5% reduces the pkHRR and THR to 37% and, 44%, respectively, compared to the control. This improved fire-retardancy for CMC_60_MTM_40_-5% stems from the thicker MTM- containing nanocomposite coating, which also leads to largely increased protective char formation on the coated textile surface (Fig. 6b–f) in accordance with TGA^54^. This thick protective solid char acts as a thermal barrier and lowers the formation of flammable volatiles, therefore, leading to the reduction in pkHRR and THR^36^,^54^,^59^. Lower pkHRR is a desired parameter to exhibit efficient fire-retardancy in real fire situation as it retards or diminishes the self-propagation or spreading of the flame to other materials when the external flame or ignition source is removed^39^. Similarly, reduction in TSR is important as smoke can cause serious problems like choking, unconsciousness, lack of breathing during evacuation^39^,^54^,^60^. A 53% reduction in TSR for CMC_60_MTM_40_-5% as compared to the control is noteworthy, indicating that not much dense smoke is released during burning of the coated fabric. Therefore, CMC_60_MTM_40_-5% appears as a very effective fire retardant nanocomposite coating.

### Surface morphology characterization

**Figure 7:**
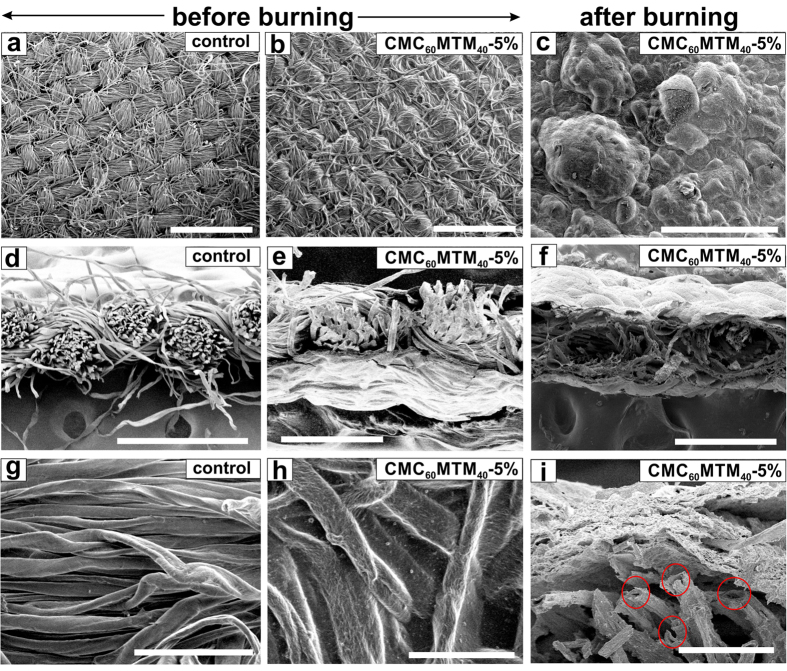
SEM images of the CMC_60_MTM_40_-5% coated fabric and the control. (**a**,**d**,**g**) Surface and cross-section of the control, and (**b**,**e**,**h**) those of CMC_60_MTM_40_-5%, before burning. (**c**,**f**,**i**) CMC_60_MTM_40_-5% after burning in VFT showing (**c**) the surface, and (**f**,**i**) the cross-section. (**i**) Individual hollow fibers are seen after burning of CMC_60_MTM_40_-5%, encircled in red. (The control was completely consumed during burning, so no image can be shown after burning) (**a**,**b**,**c**,**d**, scale bar 1 mm; (**e**,**f**) scale bar 300 μm; (**g**,**h**) scale bar 100 μm; (**i**) scale bar 50 μm).

To evaluate the surface morphology before and after burning, we further characterized selected samples by SEM (Fig. 7). The SEM images show that CMC_60_MTM_40_ coatings on textiles retain a similar weave structure as in the control (Fig. 7a,b, and Supplementary Fig. S1a). Under high magnification, the individual fibers of the control show smooth and clean surfaces (Fig. 7g) but become rougher after applying the coating (Fig. 7h, and Supplementary Fig. S1b). Moreover, for CMC_60_MTM_40_-5%, the individual fibers are linked together, and the gap between them, which may serve as sites for the penetration of heat/flames, are diminished due to a thicker coating (Fig. 7h)^36^,^52^,^53^. The SEM images after burning show that the thinner conformal coating of CMC_60_MTM_40_-1% is enough to preserve the weave structure, although considerable shrinkage is observed, creating gaps between yarns (Supplementary Fig. S1c,d). In contrast, due to thicker coating, CMC_60_MTM_40_-5% shows an intumescent effect by forming an expanded foam-like char with a lot of bubbles on its surface (Fig. 7c,f). These bubbles are formed due to evolution of gas while burning the intercalated polymer during exposure to flames. As a result, a porous foam-like structure develops that provides good heat insulation to the underlying materials^57^,^61^. A significant expansion of the coating from the initial 8.5 ± 3 to 25–50 μm enables CMC_60_MTM_40_-5% to self-extinguish quickly once the direct flame is removed and to stop the flame propagation (Fig. 7i).

Furthermore, dehydroxylation of the aluminosilicates results in self-cooling via water formation and also improves mechanical integrity due to char formation^49^. Interestingly, after burning, we also observe some hollow fibers with diameters between 7–15 μm, and hence similar to the average diameters of the original fibers. This confirms that the CMC/MTM dispersion penetrates through the textile during the coating procedure and coats the individual fibers.

### Amphiphobic and self-cleaning features

Given the diverse requirements of materials in general, especially in a real application context, it is important to develop multifunctional property profiles. One of the major bottlenecks in the application of water-borne polymer/clay nanocomposite coatings, no matter whether prepared by LbL strategy or by the faster self-assembly approach herein, is the inherent susceptibility to water^50^,^62^. Apart from fire and heat barrier properties, to promote functionality and applicability of the coated fabrics in harsh or humid environment, we sought to impart them with an amphiphobic coating, providing water (and oil) repellency and combining it with self-cleaning features. The latter is generally obtained by coatings of low surface energy and hierarchical roughness^30^,^63^,^64^,^65^,^66^.

**Figure 8:**
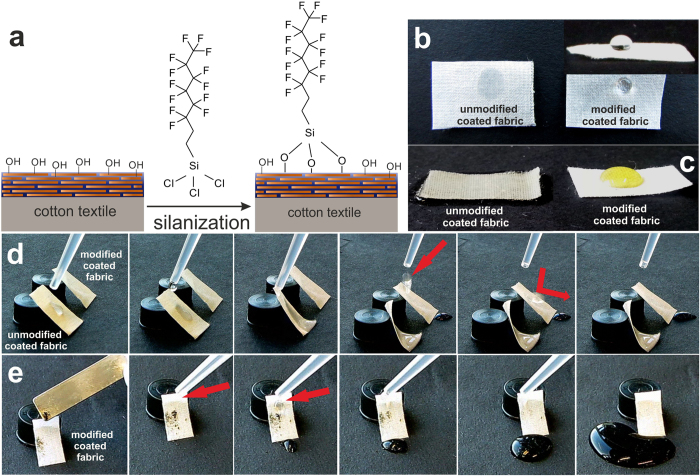
Surface modification and photographs showing amphiphobicity, water roll off and self-cleaning of the coated fabrics. (**a**) Schematic representation of surface modification of CMC_60_MTM_40_-coated fabrics with trichloro(*1H,1H,2H,2H*–perfluorooctyl)silane (not in scale). Photographs of (**b**) water droplets, and (**c**) salad oil droplets, on both unmodified and modified coated fabrics. (**d**) Water droplet is soaked on the surface of the unmodified coated fabric, whereas it can easily roll off and bounce off from the modified coated fabric. (**e**) Self-cleaning behavior of modified coated fabric.

We choose a fabric coated from intermediate slurry concentration (CMC_60_MTM_40_-2.5%) and performed a straightforward surface modification with trichloro(*1H,1H,2H,2H*–perfluorooctyl)silane (Fig. 8a), which binds to the hydroxyl groups available at the CMC. We emphasize that other amphiphobic coatings without fluorine may also be deposited given the availability of hydroxyl groups^67^,^68^,^69^,^70^,^71^. Due to the high polarity of the CMC, the unmodified coated fabric rapidly absorbs water and bends under the weight of the absorbed water. Yet, once modified with the low surface energy fluorosilane, the fabric shows hydrophobic behavior with high repellency and high contact angles towards water (Fig. 8b,d, water contact angle ca. 100°, not shown here).

At the same time, the modification also provides protection against non-polar liquids such as salad oil, a clear advantage of using fluorinated coatings over only hydrophobic coatings (Fig. 8c). The snap shot series in Fig. 8d further shows that the water droplets bounce on the surface of the modified coated fabric, and easily roll down with a low sliding angle (<25°). This is due to the low adhesiveness resulting from the cooperation of the surface hierarchical structure of the fabric and the low surface energy of fluorosilane^30^,^64^,^72^. This is a beneficial feature for self-cleaning surfaces, and prompted us to also investigate the potential for self-cleaning. Using deposited activated carbon, we find that a series of water droplets easily removes the deposited dirt and recovers the pristine clean surface (Fig. 8e). To check repeatability, we perform four cycles (1 cycle = dirt deposition/cleaning/drying) of the self-cleaning test on the same coated fabric sample and obtain pristine clean surface each time without any macroscopic damage. Surface modification of the coated fabric can further be extended to more eco-friendly and biocompatible silicon based compounds in the future^67^,^73^,^74^. Overall this demonstrates that simple surface coatings can be applied to the nacre-mimetics CMC/MTM fire barrier coatings, and amphiphobic and self-cleaning features can be imparted as desired for application needs.

## Discussion

We demonstrated a single step, large-scale, self-assembly approach to prepare thick, bioinspired, well-defined, hybrid brick-walls as fire-retardant coatings on textiles. The coating weight/thickness is tuned by changing the slurry concentrations, and the morphology can be changed from a conformal fiber coating (low concentration) to a micrometer-thick continuous coating (high concentration) on the textile. Hence, this process makes a stark difference to polymer/nanoclay multilayers formed by LbL, which could provide only conformal and ultrathin coatings (<1 μm) on fibers in alternating deposition from dilute suspensions^38^,^43^,^44^,^45^,^52^,^53^.

The fire barrier properties of the coated fabrics increase with increasing slurry concentration, making CMC_60_MTM_40_-5% the best fire barrier coated fabric. It not only withstands long-term high temperature direct flame exposure, but also prevents the penetration of flame through it by forming a shape persistent solid char during fire break-through test. It exhibits notably lower temperature build-up during VFT, and significantly decreased pkHRR (37%), THR (44%), and TSR (53%) compared to the control as seen from cone calorimetry, suggesting the generation of fewer flammable volatiles, and smoke, respectively. Furthermore, the SEM images of the post-burn chars of all coated fabrics in VFT showed that the fabric weave structure is well maintained even for CMC_60_MTM_40_-1%. CMC_60_MTM_40_-5% exhibits an intumescent effect and forms a thick and expanded foam-like char with lot of bubbles on its surface due to gas evolution. This nanoporous char provides good heat insulation to the underlying materials and improves fire retardancy.

In terms of functional benefits, we imparted amphiphobic and self-cleaning features by a simple surface modification with a fluorosilane. This furnishes a water- and oil-resistant surface based on water-borne CMC/MTM coatings, imparts self-cleaning, and thereby increases the usefulness for applications in harsher conditions.

Importantly, the presented strategy opens a generic platform towards sustainable, high-performance and non-flammable thick nanocomposite coatings. As a major advantage, these fire-retardant coatings are devoid of any halogen atoms or heavy metals, often required in present day fire-barrier materials, and processed from water using sustainable building blocks. We expect that this continuous roll-to-roll coating process can be scaled to large quantities and implemented into existing coating technology. While we showcased the applicability on textiles, we believe it to be applicable on diverse substrates, and that further tuning of type of polymer system will allow an optimization of the properties and increased intumescent behavior. Further studies on these systems are currently under way.

## Methods

### Materials

Sodium carboxymethyl cellulose (CMC, degree of substitution = carboxymethyl groups per anhydroglucose unit = 0.7, M_w_ = 90 kg/mol, Aldrich), Na-Cloisite (MTM, Rockwood), Trichloro(*1H,1H,2H,2H*–perfluorooctyl)silane (97%, Aldrich), MilliQ water, and pure cotton textile (ISO 105-F, 150 mm width, 110 g/m^2^ density) were used for all experiments.

### Preparation of bioinspired hybrid fire-retardant nanocomposite coating on textile

CMC and MTM were added as premixed (60/40 w/w) powder into a IKA HKD-T-0.6 high-performance kneader with two wide-bladed kneading elements containing the appropriate amount of water to reach a total concentration of ca. 8 wt%. After homogenization for 12 h, the slurry was diluted to different concentrations (1, 1.75, 2.5 and 5 wt%), and degassed. Then a custom-made continuous coating machine was used to guide the cotton textile (width 150 mm, density 110 g/m^2^) from a roll through the coating bath at 10 mm/min for each slurry concentration. Afterwards, the fabrics were dried at ambient conditions. We use the following nomenclature for the coating compositions. Textile coated with CMC/MTM = 60/40 w/w at various concentrations are abbreviated as CMC_60_MTM_40_-x%, where x = 1, 1.75, 2.5 or 5 wt%.

### Surface modification of bioinspired hybrid fire-retardant nanocomposite coating on textile

A CMC_60_MTM_40_-2.5% coated textile was dipped into a 5 mM solution of trichloro(*1H,1H,2H,2H*–perfluorooctyl)silane in heptane, and left for 15 min. Afterwards, the coated textile was rinsed with heptane and water few times and dried.

**Field emission-scanning electron microscopy (SEM)** was performed on a Hitachi S-4800 field emission microscope using 1.5 kV acceleration voltage.

**Thermogravimetric analysis (TGA)** was done using a NETZSCH TG 209 C instrument in both air and N_2_ atmosphere from 25 to 850 °C at a heating rate of 10 °C/min.

**Wide-angle x-ray diffraction (WAXD)** was performed using an Empyrean setup from PANalytical using the Bragg Brentano parallel-beam geometry. An Empyrean Cu x-ray tube LFF HR (line source of 12 × 0.04 mm^2^) provided CuKα_α_ radiation with *λ* = 1.542 Å at 40 kV voltage and 40 mA current.

**Fire break-through tests** were performed on the coated fabrics and the uncoated control using a Soudogaz^®^ X 2000 PZ gas torch with maximum flame temperature of ca. 1750 °C. All samples were conditioned for 1–2 days at 23 ± 2 °C and 50 ± 5% relative humidity (RH) prior to test. Then samples were cut into circular shape having ca. 7 cm in diameter and fixed in a circular metal holder keeping a 130 mm distance between the samples and the origin of the flame. The samples were exposed to the direct flame at an angle close to 90° and the fire break-through process was monitored using a video camera.

**Bench-scale vertical flame tests (VFT)** were performed on the coated fabrics and the uncoated control with dimensions of 160 mm × 12.5 mm (L × W) in analogy to EN ISO 11925-2. The samples were conditioned for 1–2 days in normal climate at 23 ± 2 °C temperature and 50 ± 5% RH prior to test. A yellow flame was kept at the bottom of the sample for 5 s. The burning was monitored using a forward looking infrared camera A655sc camera (FLIR) in the temperature range of 20–660 °C, and standard video.

**Cone calorimeter experiments** were conducted on a FTT cone calorimeter at 50 kW/m^2^ heat flux, with an exhaust flow of 24 L/s, according to ISO 5660, on sample dimensions of 100 mm × 100 mm (L × W) conditioned for 24 h at 23 °C and 50% RH prior to test. The heat release rate was determined by the measurement of the oxygen consumption derived from the oxygen concentration and the flow rate in the combustion product stream. Parameters such as time to ignition (TTI), peak heat release rate (pkHRR), total heat release (THR), and total smoke release (TSR) were evaluated.

## Additional Information

**How to cite this article**: Das, P. *et al*. Large-scale, thick, self-assembled, nacre-mimetic brick-walls as fire barrier coatings on textiles. *Sci. Rep.* **7**, 39910; doi: 10.1038/srep39910 (2017).

**Publisher's note:** Springer Nature remains neutral with regard to jurisdictional claims in published maps and institutional affiliations.

## Supplementary Material

Supplementary Information

## Figures and Tables

**Table 1:** Overview from cone calorimetry measurement.

Sample	TTI (s)	pkHRR (kW/m^2^)	avg. HRR (kW/m^2^)	THR (MJ/m^2^)	TSR (m^2^/m^2^)
control	8 ± 2	88 ± 1	28 ± 2.5	1.8 ± 1	4.5 ± 1
CMC_60_MTM_40_ ─ 1%	8 ± 2	73 ± 3	28 ± 1.5	1.7 ± 0.1	3.6 ± 0
CMC_60_MTM_40_ ─ 1.75%	9 ± 3	70 ± 2	25 ± 2.1	1.6 ± 0.1	3.8 ± 0.6
CMC_60_MTM_40_ ─ 2.5%	5 ± 1	69 ± 3	27 ± 0.6	1.7 ± 0.1	1.0 ± 0.5
CMC_60_MTM_40_ ─ 5%	7 ± 2	55 ± 7	17 ± 2.2	1 ± 0.1	2.1 ± 1.1

TTI = time to ignition, pkHRR = peak heat release rate, avg. HRR = average heat release rate, THR = total heat release rate, TSR = total smoke release.
