# Supplementary material for: Large-scale, thick, self-assembled, nacre-mimetic brick-walls as fire barrier coatings on textiles
Source: Sci Rep. 2017 Jan 5;7:39910. doi: 10.1038/srep39910 (PMC5215295; doi:10.1038/srep39910)
Supplement: Supplementary Information [file srep39910-s1.pdf]

Supplementary Information for:

Large-scale, thick, self-assembled, nacre-mimetic brick-walls as fire barrier coatings on textiles

Paramita Das, Helga Thomas, Martin Moeller, Andreas Walther*

DWI – Leibniz-Institute for Interactive Materials, Forckenbeckstr. 50, 52056 Aachen, Germany

Correspondence and requests for materials should be addressed to A.W.

(email: walther@dwi.rwth-aachen.de).

Content:

1. SEM images of the $CMC_{60}MTM_{40}$-1% coated fabric 2

1. SEM images of the CMC60MTM40-1% coated fabric

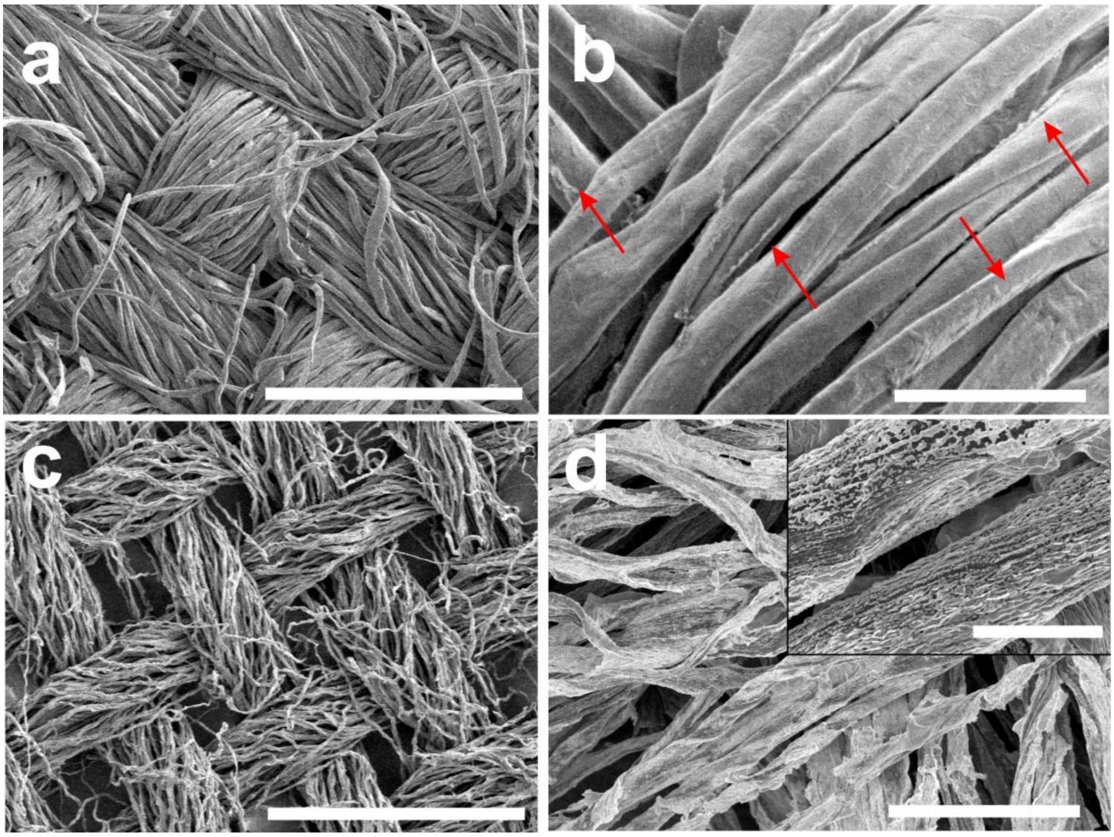

Supplementary Figure S1. SEM images of the CMC60MTM40-1% coated fabric. (a-d) Surface of CMC60MTM40-1%, (a,b) before burning, and (c,d,inset) after burning. The arrows guide the eye to locate rougher areas of the conformal coating of individual fibers (a,c, scale bar 500 µm; b,d, scale bar 50 µm, inset of d, scale bar 5 µm).
